# Initiative for Promoting Affordable and Quality Tuberculosis Tests (IPAQT): a market-shaping intervention in India

**DOI:** 10.1136/bmjgh-2019-001539

**Published:** 2019-11-28

**Authors:** Harkesh Dabas, Sarang Deo, Manisha Sabharwal, Arnab Pal, Sachin Salim, Lakshmi Nair, Kaartikeya Chauhan, Prateek Maheshwari, Aparna Parulkar, Ritu Singh, Manasi Chitalia, Rigveda Kadam, Manjot Kaur, Collins Oghor, Nirros Ponnudurai, Sameer Kumta, Peter Small, Puneet Dewan, Madhukar Pai

**Affiliations:** 1 Clinton Health Access Initiative, New Delhi, India; 2 Operations Management, Indian School of Business, Hyderabad, India; 3 Epidemiology and Biostats, McGill University, Montreal, Quebec, Canada; 4 Bill and Melinda Gates Foundation, New Delhi, India

**Keywords:** health systems, public health, tuberculosis, intervention study

## Abstract

A majority of patients with tuberculosis (TB) in India are diagnosed and treated in the private sector. Yet, most private providers do not use most recent WHO-endorsed microbiological tests such as liquid cultures, line probe assays and Xpert MTB/RIF due to a combination of factors such as lack of awareness, misaligned incentives and high prices that are unaffordable for patients. We designed a market-based approach to transform a high-price, low-volume market equilibrium into a low-price, high-volume equilibrium to improve the uptake of these tests. Toward this end, a non-profit consortium of private laboratories, called Initiative for Promoting Affordable and Quality Tuberculosis Tests (IPAQT) was formed in India in March 2013. It negotiated lower pricing on equipment and reagents with manufacturers, closer to that offered to the public sector. In return, IPAQT assured that this discount was passed on to patients, who typically paid for these tests out of their pockets, through an informally agreed on retail ceiling price. IPAQT also invested in demand generation activities that complemented the supply-side effort. IPAQT membership grew from 56 laboratories in 2013 to 211 in 2018. During this period, the initiative resulted in a 10-fold increase in the uptake of Xpert and a 30%–50% reduction in price. This initiative is planned to be expanded to other South Asian countries with similar TB epidemic and private market structure and dynamics. However, long-term sustainability of the initiative would require developing more cost-effective marketing activities and integration with broader private sector engagement agenda of the national TB programme.

**Summary box**

- A significant fraction of patients with tuberculosis (TB) in high burden countries are diagnosed and treated by private healthcare providers, whose uptake of WHO-endorsed TB tests is suboptimal due to low awareness and weak economic incentives driven by lack of affordability among their patients.
- A market-based initiative was developed in India wherein a consortium of private laboratories negotiated lower pricing from manufacturers and distributors and, in turn, agreed to provide lower ceiling price for patients.
- Over a period of 5 years, uptake of WHO-endorsed tests increased more than 10-fold, along with a reduction in commercial price of the tests outside of the consortium.
- Financial sustainability of this initiative is likely to require integration with larger government efforts to engage with private providers, expansion to cover more tests and leveraging digital technology to increase awareness among private providers at scale.

## Background

The private sector is a major provider of healthcare in many countries with high burden of tuberculosis (TB).^1^ In these countries, patient pathways (including those for low-income segments) comprise visits to multiple providers leading to delayed diagnosis and suboptimal quality of care.^2–4^ Thus, large-scale engagement of private providers, including rapid scale-up of novel TB diagnostic tools, is critical for TB elimination by 2030^1 5–7^

In India, more than half of the estimated 2.8 million patients (>25% of the global TB burden) are treated in a highly fragmented private sector with poor diagnostic and treatment practices.^8–10^ Most private providers diagnose TB based on a combination of chest X-ray, non-specific laboratory tests and empiric treatment instead of WHO-recommended sputum-based microbiological tests.^9 11–13^ They also used antibody-based serological tests, despite the lack of clinical accuracy, until they were nationally banned in 2012.^14–17^ In contrast, WHO-endorsed tests such as Xpert MTB/RIF, line probe assays (LPAs), and liquid cultures experienced limited uptake due to a combination of high-input pricing (compared with pricing for public and non-profit sectors), import duties, distributor and laboratory profits and physician incentives combined with limited willingness or ability of patients to pay for them out of pocket.

## Overall approach

We designed a market-based approach on the premise of existence of a large potential market for TB tests given the high TB burden in India and high use of private health services including laboratories. In particular, we attempted to increase the adoption of WHO-endorsed tests in the Indian private health sector by transforming a high-price, low-volume market equilibrium into a low-price, high-volume equilibrium. The underlying theory of change was *lower prices for high-quality tests in the private sector, combined with increased awareness of their benefits, will result in increased testing of patients with presumptive TB and sustained private sector uptake, provided that the earnings for all stakeholders in the diagnostic value chain (manufacturers, distributors, laboratories and providers) were protected and potentially enhanced by higher volumes*.

**Figure 1:**
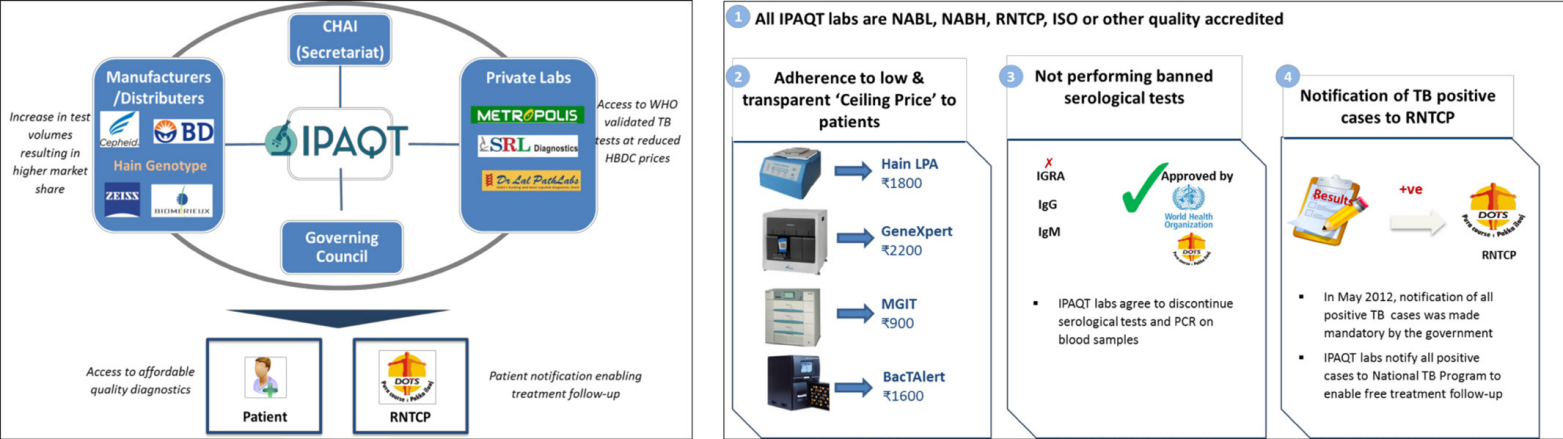
Governance structure and guiding principles of IPAQT. CHAI, Clinton Health Access Initiative; IPAQT, Initiative for Promoting Affordable and Quality Tuberculosis Tests; ISO, International Standards Organization; NABH, National Accreditation Board for Hospitals; NABL, National Accreditation Board for Testing and Calibration Laboratories; TB, tuberculosis; RNTCP, Revised National Tuberculosis Control Program.

Toward this end, a non-profit consortium of private laboratories called Initiative for Promoting Affordable and Quality TB Tests (IPAQT) was launched in March 2013 (figure 1). The governing council of the non-profit consortium comprised heads of select private laboratories, whereas the secretariat was managed by a not-for-profit entity, the Clinton Health Access Initiative. Funding for the secretariat was provided by international funding agencies, namely, the Bill & Melinda Gates Foundation and Department for International Development (DFID). Technical and monitoring and evaluation support was provided by international research institutions, namely, McGill International TB Centre and Indian School of Business.

All IPAQT member laboratories agreed to abide by the following guiding principles: (1) have quality accreditation, (2) offer only high-quality (WHO-endorsed) TB tests, (3) notify patients with TB to the governmental health authorities and (4) comply with predetermined ceiling prices for tests. The secretariat undertook several supply-side and demand-side activities to facilitate implementation of these principles and increase adoption of quality TB tests, thereby creating a ‘win–win–win’ platform for all players in the value chain.

### Supply-side activities

Before IPAQT, WHO-endorsed TB tests were supplied to the public sector in high-TB burden countries at prices that were significantly lower (the so-called ‘High Burden Developing Country (HBDC) price’) than those supplied to the private health sector providers. IPAQT negotiated with manufacturers (including BD, Cepheid and Hain LifeScience) to obtain concessional pricing (30%–50% discount on existing commercial rates) on equipment and reagents for member laboratories, comparable with price offered to the public sector in high-TB burden low-income countries. In return, IPAQT provided assurance that this discount would be passed on to patients, who typically paid for these tests out of their pockets, and accordingly set the retail ceiling price. The manufacturers found this move to be incentive-compatible as the private market for these tests was small and the likelihood of private demand picking up at prevalent prices was low. Following the agreements with the manufacturers, margins of other channel partners (eg, distributors), including informal incentives between these stakeholders, were also reduced proportionally to support the lower retail price. Most distributors handled multiple diagnostic tests. Hence, they faced limited downside risk if the volume did not increase even after the reduction in the margin. The resulting retail prices for WHO-endorsed TB tests were approximately 50% lower for Xpert MTB/RIF (US$67–US$33) and Hain Line Probe Assay (US$58–US$27) and approximately 15% lower for MGIT Liquid Culture (US$18–US$15).

### Demand-side activities

IPAQT implemented several strategies to increase demand for quality TB tests among private providers. These were adapted from marketing and sales practices in the pharmaceutical industry and included continuing medical education (CME) seminars, focused group discussions (FGDs) and in-person visits by field sales force.

More than 120 CME seminars were conducted in 35 cities between 2013 and 2016 by national and international experts to educate providers on WHO-endorsed diagnostic tests. Local IPAQT member laboratories were responsible for marketing these events among high potential providers in the city.

A field sales force with prior pharmaceutical sales experience was deployed between October 2014 and June 2016 in seven cities: Mumbai, Delhi, Patna, Coimbatore, Lucknow, Ahmedabad and Pune. It made routine visits to private providers and informed them on technical attributes of the tests, their availability at nearest IPAQT network laboratories and Standards of TB Care in India. In 2017, FGDs moderated by key opinion leaders were introduced to promote greater interaction and engagement among the participants.

Above routine activities were complemented by small experiments aimed at monitoring, learning and evaluation. Key examples include a randomised controlled trial to test the effectiveness of providing free samples (ie, vouchers for free Xpert tests) to providers in increasing their long-term adoption of tests and an external quality assurance study to assess the quality of Xpert MTB/RIF testing.

## Network expansion and market coverage

**Figure 2:**
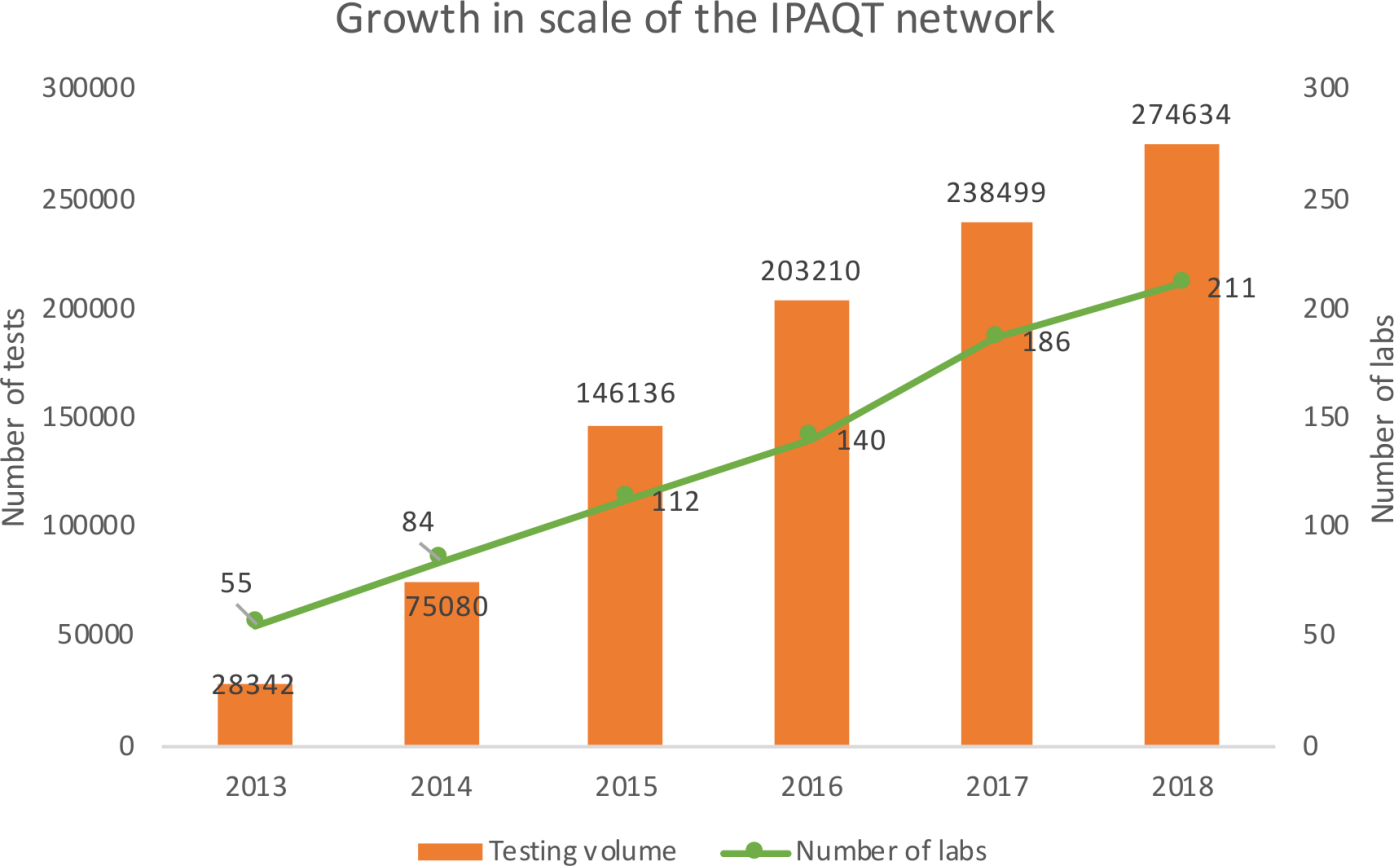
Growth in testing volume and number of laboratories in the IPAQT network. IPAQT, Initiative for Promoting Affordable and Quality Tuberculosis Tests.

IPAQT membership grew from five laboratories in 2013 to 211 in 2018 (figure 2) with >6000 sample collection centres across 380 districts. These included seven national chains, 30 regional chains, 103 stand-alone laboratories and 69 hospital/medical college laboratories. Based on more than 30 responses to an online survey of member laboratories, we found ‘access to newest technology at competitive prices’ and ‘social responsibility’ as the most common reasons cited for joining IPAQT. However, IPAQT laboratories comprise only ~20% of National Accreditation Board for Testing and Calibration Laboratories accredited private (national-chain, regional-chain and stand-alone) laboratories conducting microbiological testing laboratories and ~20% of National Accreditation Board for Hospitals accredited private hospitals in tier II and III cities (cities with relatively lower population and lower cost of living). (This classification is based on the house rent allowance provided by the central.)

## Trends in test volumes

**Figure 3:**
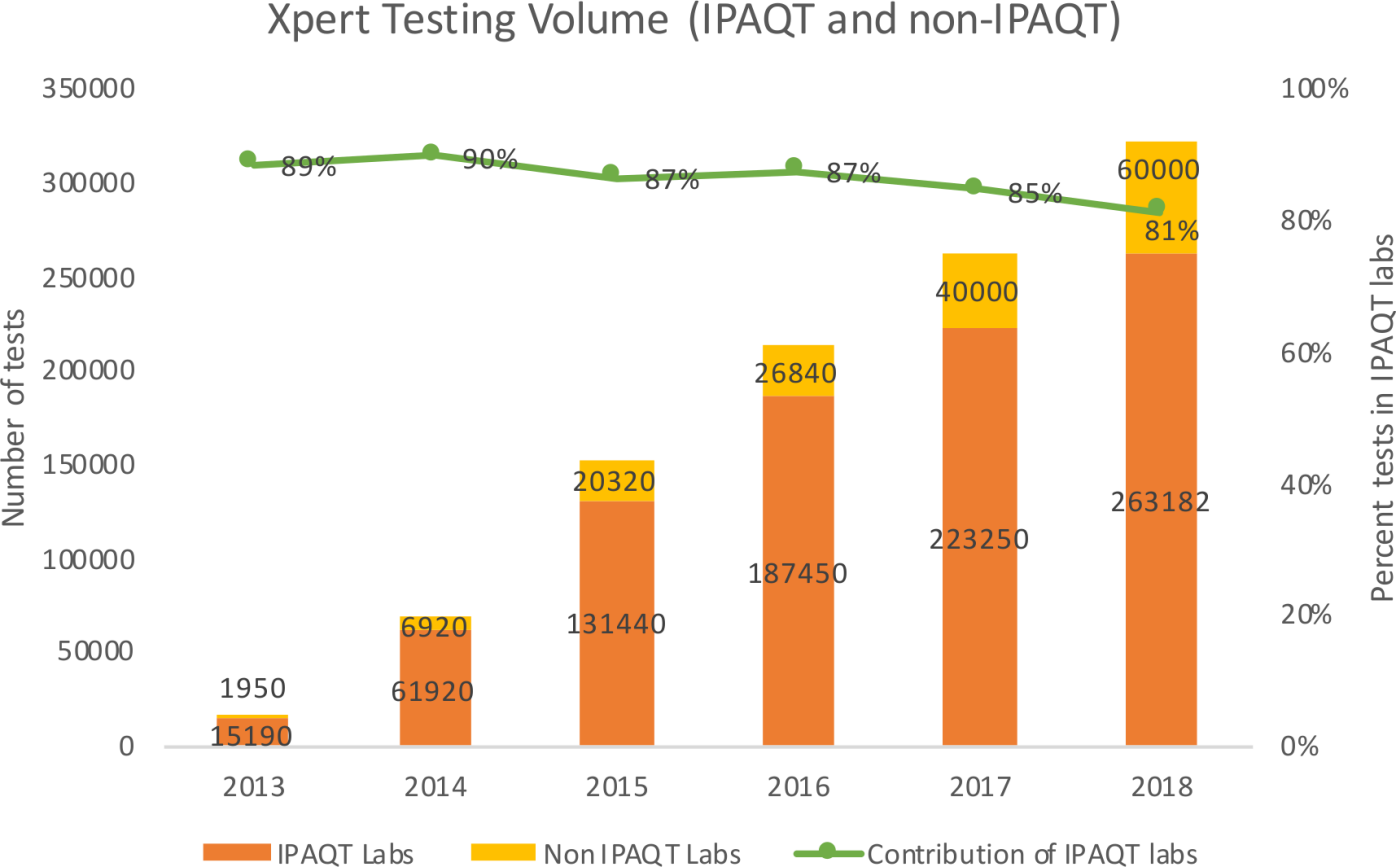
Contribution of IPAQT to Xpert MTB/RIF testing volume in India's private sector. IPAQT, Initiative for Promoting Affordable and Quality Tuberculosis Tests.

IPAQT laboratories conducted around 620 000 Xpert MTB/RIF, 47 000 LPAs and 25 000 BacTAlert Liquid Culture tests from 2013 to 2018. For all WHO-endorsed tests combined, testing volume increased from ~28 000 tests in 2013 to ~2 75 000 tests in 2018 (figure 2). Overall, national chain laboratories contributed 45% of IPAQT GeneXpert and LPA volumes. Three national chains, one regional chain and one hospital contributed ~60% of the total volume. Xpert MTB/RIF contributed >80% of test volume due to its ease of use, quick turn-around time, drug resistance markers and relatively lower technical requirements. Based on data obtained from Cepheid, we estimated that IPAQT contributed more than 80% of total Xpert volume in the private sector in India (figure 3). It is worth noting that this contribution has reduced slightly from 89% in 2013 to 81% in 2018 owing to rapid growth in Xpert testing volume in non-IPAQT laboratories. Moreover, compared with >400 000 Xpert tests conducted by IPAQT laboratories in India during 2014–2016, sales in eight other high-burden countries were ~156 000 tests (table 1).

**Table 1:** Sales of Xpert MTB/RIF in the private sector in countries other than India (source: Cepheid)

Country	2014	2015	2016	2017 (until October)
Myanmar	1910	150	280	20
Indonesia	200	50	–	50
Malaysia	950	2910	3200	3727
Philippines	2760	8820	14 348	7860
Singapore	1250	8178	12 547	10 000
South Korea	25 288	32 971	39 441	40 099
Vietnam	40	80	150	180
Papua New Guinea	–	200	400	1400
Grand total	32 398	53 359	70 366	63 336

## Trends in test prices

**Figure 4:**
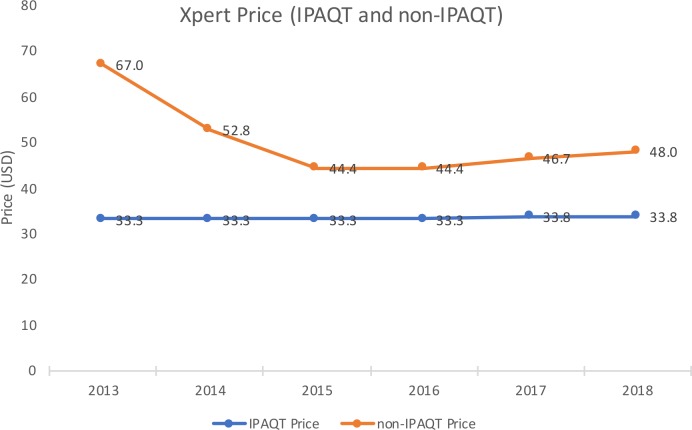
Comparison of IPAQT and commercial prices for Xpert MTB/RIF in India’s private sector. IPAQT, Initiative for Promoting Affordable and Quality Tuberculosis Tests.

Based on data compiled from non-IPAQT laboratories, we found that IPAQT price of Xpert MTB/RIF ($33.8) was consistently lower than non-IPAQT private market price ($46.7) over the intervention period (figure 4). Interestingly, non-IPAQT price also experienced a substantial reduction from 2013 to 2017. This suggests that the lower pricing offered by IPAQT laboratories, which are dominant players in the market, may have created a downward pressure on these commercial prices. These prices were also lowest among seven countries with a comparably sized private healthcare sector.^7^ Furthermore, the average price in those countries increased from $68.73 in 2015 to $84.53 in 2017 compared with the downward trend observed in India. We could not conduct a similar analysis for other tests as we did not have access to commercial pricing data for those tests from their manufacturers.

## Lessons learned

Our results demonstrate the effectiveness and point to certain limitations of a market-based approach, in particular, the ‘low-margin high-volume’ strategy, to improve access to affordable and high-quality diagnostic tests among large and fragmented private sectors.

IPAQT was designed to be systematically different from previous interventions aimed at reducing prices of global health commodities, most notably the Global Drug Facility (GDF), due to key contextual factors. First, IPAQT was developed and implemented at a national level and hence had a much smaller scale compared with GDF, which is a multinational initiative. Second, on the supply side, a large part of price reduction was achieved by GDF through competitive tendering by suppliers. This was not possible in the IPAQT model due to the proprietary nature of each test. Instead, price reduction was achieved by the prospect of larger volumes, as well as lowering the cost for patients by guaranteeing that laboratories pass the benefits to the patients. Third, GDF’s demand side partners were national TB programmes, which made uptake of the drugs and tests easier as the cost of these tools was not borne by patients. In contrast, IPAQT was implemented in the private sector and patients paid for the tests out-of-pocket. As a result, IPAQT had to make substantial investment in aggregating private laboratories, as well as demand generation activities to complement the supply-side efforts.

Despite achieving a 10-fold increase in volumes of WHO-approved TB tests across India during a 5-year period, current testing volume under IPAQT (~275 K tests) represents a small fraction of the potential demand; there are estimated 2.2 million patients with TB per year in India’s private sector,^8^ which roughly translates to 22 million patients with presumptive TB who should be evaluated every year. Three main challenges underlie IPAQT’s limited success with regard to market penetration. First, there are extensive data showing serious undertesting for TB in the private health sector and great reluctance of private providers to order sputum-based TB tests.^9 11 13^ Indeed, standardised patient studies in India consistently show underuse of diagnostic tests and overuse of medications, especially antibiotics.^9–11^ Second, IPAQT’s funding allowed limited investment in demand-side activities, which tend to be highly effort intensive and resource intensive due to the fragmented base of private healthcare providers (laboratories and physicians). This was further exacerbated by the lack of technical marketing skills and resources at many smaller laboratories required to drive adoption of new technologies among uninformed providers. Third, even the reduced price of ~$30 for Xpert MTB/RIF, the most important new WHO-endorsed tool, is prohibitively high for a large majority of patients in India, especially those from low-income segments that comprise a large majority of India’s patients with TB. These patients have higher perceived values for tangible medical interventions, such as antibiotics, steroids and intravenous fluids, that provide immediate symptomatic relief compared with diagnostic tests that only provide information.^13^

## Policy implications for sustainability

The key to overcoming the prviously mentioned challenges and ensuring long-term sustainability of the IPAQT model depends on making the demand generation efforts more cost-effective. A few initiatives have been launched in this pursuit. From 2017, a digital marketing approach, which is less resource intensive compared with a field force model, was piloted, wherein information regarding WHO-endorsed TB tests was disseminated to private providers through a digital platform via mobile phones. It involved conventional content, such as articles and videos by experts and peers, combined with innovative interactive elements, such as gaming. Further, from early 2018, the IPAQT model was expanded to cover HCV and HIV viral load testing at lower pricing to improve the utilization of the existing capacity of testing platforms such as GeneXpert and to ensure that the marketing and capital equipment costs are apportioned over multiple tests, thereby improving financial sustainability of those activities. Until January 2019, 76 private laboratories had enrolled for HCV and HIV viral load testing under IPAQT.

Finally, IPAQT can be integrated within the broader efforts of private sector engagement that have shown the ability to change provider and patient behaviours at pilot scale in Mumbai and Patna^18^ and are being scaled up under the ambitious project, Joint Effort for Elimination of TB, launched with funding from The Global Fund. Under this project, Patient Provider Support Agencies (PPSAs) are engaging with private providers to improve the quality of TB diagnosis and treatment in 45 cities across 23 states. In addition to facilitating free testing of patients with presumptive TB in public sector laboratories, PPSAs will also disseminate information on IPAQT laboratories to willing patients who prefer and can afford paid tests in the private sector. Encouraged by these results, the IPAQT model is currently in the process of being piloted in other countries in South Asia (eg, Pakistan) and Africa, which have private markets similar to that of India.

## Limitations

Our analysis capturing the impact of IPAQT has limitations owing to the design of the intervention and the associated data sources. First, IPAQT is a service-delivery project, not a research study. It was not designed as a randomised intervention. As a result, it was not possible interpret the difference in change in testing volumes between IPAQT and non-IPAQT laboratories as a causal effect of the intervention. In other words, laboratories that agreed to join the intervention were probably systematically different (eg, larger, accredited) from those who did not join. Although we conducted a survey of IPAQT laboratories to elicit their reasons for joining the intervention, the response rate was very low, which limits the generalisability and representativeness of the findings.

More fundamentally, IPAQT is a market-level (and not a laboratory-level) intervention. Hence, a rigorous evaluation would have required randomising different markets for TB diagnostic tests into control and intervention arms. Given the presence of large national laboratories, this would have meant launching the initiative in multiple countries, which was beyond the scale and scope of available resources.

As a corollary to the design of the intervention, we used data obtained from the intervention partners, that is, manufacturers and laboratories, to assess its impact. Consequently, we had to exclude some tests and laboratories from our assessment, which were either not part of the intervention or were unwilling to share their proprietary data for analysis and dissemination.

Finally, our insights regarding the effectiveness of IPAQT models are naturally restricted to diagnostic tests that require laboratory testing. An increasing number of point-of-care (POC) tests are being developed and commercialised (eg, lateral flow urine lipoarabinomannan (LAM) test), which can be conducted at the healthcare facility itself. The economics (eg, margins across different value chain entities) for these POC tests is likely to be very different compared with tests that require centralised laboratory processing. As a result, any market-making intervention may need to be designed differently.

## Conclusion

Our results indicate the feasibility and effectiveness of a novel, market-based intervention for improving access to essential health commodities in private sector in low-income and middle-income countries, where out-of-pocket spending by patients is a dominant source of healthcare financing. Such intervention is able to move private, for-profit entities from a low-volume, high-price equilibrium to a high-volume, low-price equilibrium through simultaneous demand-side (marketing and demand generation) and supply-side (modification of margins of various supply chain entities) activities. It is also feasible to expand TB-specific market interventions to include other related diseases, and this is significant within the context of universal health coverage (UHC). Long-term sustainability of such intervention will depend on the ability to integrate it with broader private sector engagement programmes and UHC investments and programmes from national and state governments.
